# Enhancing genetic association power in endometriosis through unsupervised clustering of clinical subtypes identified from electronic health records

**DOI:** 10.21203/rs.3.rs-5004325/v1

**Published:** 2024-09-09

**Authors:** Lindsay A Guare, Leigh Ann Humphrey, Margaret Rush, Meredith Pollie, James Jaworski, Alexis T Akerele, Yuan Luo, Chunhua Weng, Wei-Qi We, Leah Kottyan, Gail Jarvik, Noemie Elhadad, Penn Medicine Biobank, Regeneron Genetics Center, Krina Zondervan, Stacey Missmer, Marijana Vujkovic, Digna Velez-Edwards, Suneeta Senapati, Shefali Setia-Verma

**Affiliations:** 1 Genomics and Computational Biology, University of Pennsylvania, Philadelphia, Pennsylvania, United States of America; 2 Department of Pathology and Laboratory Medicine, University of Pennsylvania, Philadelphia, Pennsylvania, United States of America; 3 Department of Obstetrics and Gynecology, Hospital of the University of Pennsylvania, Philadelphia, Pennsylvania, United States of America; 4 Vanderbilt Genetics Institute, Vanderbilt University, Nashville, Tennessee, United States of America; 5 Division of Epidemiology, Department of Medicine, Vanderbilt University Medical Center, Nashville, Tennessee, United States of America; 6 School of graduate studies, Department of Microbiology, Immunology, and Physiology, Meharry Medical College, Nashville, Tennessee, United States of America; 7 Division of Quantitative Science, Department of Obstetrics and Gynecology, Vanderbilt Genetics Institute, Vanderbilt University Medical Center, Nashville, Tennessee, United States of America; 8 Feinberg School of Medicine, Northwestern University, Evanston, Illinois, United States of America; 9 Department of Biomedical Informatics, Columbia University, New York City, New York, United States of America; 10 Department of Biomedical Informatics, Vanderbilt University, Nashville, Tennessee, United States of America; 11 Cincinnati Children’s Hospital Medical Center, Cincinnati, Ohio, United States of America; 12 Division of Medical Genetics, University of Washington, Seattle, Washington, United States of America; 13 Department of Genomic Epidemiology, University of Oxford, Oxford, England; 14 Department of Obstetrics, Gynecology, and Reproductive Biology, Michigan State University, East Lansing, Michigan, United States of America; 15 Department of Medicine, University of Pennsylvania, Philadelphia, Pennsylvania, United States of America

## Abstract

Endometriosis is a complex and heterogeneous condition affecting 10% of reproductive-age women, and yet, it often goes undiagnosed for several years. Limited observed heritability (7%) of large genetic association studies may be attributable to underlying heterogeneity of disease mechanisms. Therefore, we conducted this study to investigate genetic associations across sub-phenotypes of endometriosis. We performed unsupervised clustering of 4,078 women with endometriosis based on known endometriosis risk factors, symptoms, and concomitant conditions. The clusters were characterized by examining electronic health record (EHR) data and comprehensive chart reviews. We then performed genetic association for each cluster with 39 endometriosis-associated loci (Total N_endometriosis cases_ = 12,350). We identified five sub-phenotype clusters: (1) pain comorbidities, (2) uterine disorders, (3) pregnancy complications, (4) cardiometabolic comorbidities, and (5) HER-asymptomatic. Bonferroni significant loci included *PDLIM5* for the cluster 1, *GREB1* for cluster 2, *WNT4* for cluster 3, *RNLS* for cluster 4, and *ABO* for cluster 5. The difference in associations between the groups suggests complex and varied genetic mechanisms of endometriosis and its symptoms. This study enhances our understanding of the clinical patterns of endometriosis sub-phenotypes, showcasing the innovative approach employed to investigate this complex disease.

## Introduction

Endometriosis, a complex gynecological condition affects 10% of women of reproductive age globally and more than 50% of women with infertility ^1^, yet it often goes either undiagnosed or misdiagnosed, leading to delayed diagnoses and delivery of effective therapy ^2,3^. Endometriosis is characterized by a variety of symptoms that can significantly impact the quality of life of affected individuals. While the most common symptom of endometriosis is pelvic pain, endometriosis is formally characterized by the presence of endometrial-like tissue outside of the uterus via surgical procedure. Importantly, endometriosis is not just a disease of pelvis, but instead a chronic systemic disease affecting the entire human body ^4^. Emerging evidence suggests that endometriosis causes changes in metabolism and gene expression in the central nervous system, leading to low body mass index (BMI) and neurological depression symptoms ^5^. The extent of disease observed during surgery or via imaging does not necessarily correlate with reported symptoms (or vice versa). Instead, complex pathophysiology contributes to differences in the clinical and biological manifestations of endometriosis. The diagnosis of endometriosis relies heavily on clinical presentation, and traditional diagnostic strategies consider only patients who present with typical symptoms ^6^, overlooking women with atypical or distant manifestations. Understanding and quantifying this pathological heterogeneity is critical to design noninvasive, precise diagnostic tools that will enable accurate diagnoses and personalized treatment options. Electronic Health Records (EHRs) represent a rich, yet underutilized, data source for capturing the phenotypic spectrum of endometriosis ^7^. Although the symptoms for endometriosis can be quite severe, including chronic debilitating pain, dyspareunia, and infertility, the average time to diagnosis is 4.5 years ^8^, in part because the only way to definitively diagnose endometriosis is by surgical observation of endometrial lesions growing outside of the uterus (e.g. abdominal cavity, pelvis, ovaries, etc.) ^9^. The variability in symptoms and disease presentation adds to the difficulty of diagnosis and hinders the optimal use of electronic health records (EHRs) in research for accurately identifying affected individuals and control subjects ^10–12^, which is critical for understanding the disease and advancing treatment strategies. The depth and breadth of EHR data provide a unique opportunity to apply unsupervised learning techniques for the identification of distinct phenotypic clusters that may correspond to clinical subtypes of endometriosis.

Despite the prevalence and severity of endometriosis, etiology of endometriosis is still poorly understood. The pursuit thus far of biomarkers and drug targets based on genetic contributions of disease in patients with endometriosis has mainly included genome-wide association studies to identify genetic variants contributing to the disease ^13,14^. Twin studies have estimated the heritability of endometriosis to be 47.5% ^15^, and common variants are estimated to contributed 26% of phenotypic variance ^16^, but the largest GWAS to-date (N > 750,000, 60,674 cases) has only explained 7% of the phenotypic variance ^14^. Although large-scale genomic studies have promised insights into the underlying genetic mechanisms of endometriosis, yet the heterogeneity of the disease presentation has consistently complicated these efforts. Traditional genetic association studies have struggled to untangle the intricate web of genotypic and phenotypic diversity within endometriosis patients, leading to a critical need for innovative approaches to dissect the disease’s complexity.

The complex nature of endometriosis, with its diverse symptoms and overlapping features with other gynecological diseases, presents challenges for understanding its genetic mechanisms. We hypothesized that underlying clinical heterogeneity is obscuring the genetic mechanisms and preventing large-scale genetic studies from explaining more of the heritability. Building on the premise that a more nuanced understanding of endometriosis subtypes could unlock new genetic associations, our study leverages unsupervised, phenotypic clustering analysis of EHR data to systematically identify and characterize clinical subtypes of endometriosis. By dissecting the heterogeneity inherent in the disease, we aim to increase the power of genetic association analyses, facilitating the identification of subtype-specific disease mechanisms. This approach not only promises to enhance our understanding of endometriosis genetics but also to refine diagnostic criteria and inform more targeted and effective treatment strategies.

## Results

### Derivation, study, and validation datasets

This study utilized six total EHR-linked biobank datasets to investigate the genetic mechanisms underlying endometriosis and its sub-phenotypes. The datasets used were endometriosis cases in the non-genotyped PMBB for the derivation of clusters, a chart-reviewed endometriosis cohort to help characterize the clusters, the genotyped PMBB, six sites within the eMERGE network, AOU, UKBB, and BioVU for genome-wide association analyses (See Methods). The sample sizes for each cohort, the numbers of cases and controls, and the mean age at the time of data pull for each cohort are shown in Table 1. See Methods for details on each of the four datasets. By leveraging these datasets, the study aimed to identify endometriosis sub-phenotypes and gain insights into their associated genetic factors

### Derivation of unsupervised of clusters

**Figure 1.**
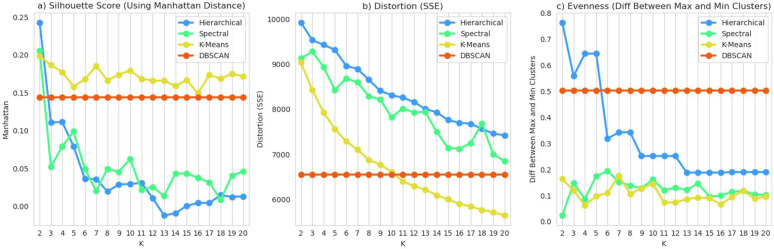
Testing unsupervised clustering methods. We tested various clustering algorithms and K-values to empirically choose an optimal method. The three metrics shown are (a) Manhattan-distance-based silhouette score, (b) distortion or sum of squared errors, and (c) evenness represented by the difference in fraction between the largest and smallest clusters. Based on these tests, we chose spectral clustering with K=5.

Unsupervised clustering was performed in non-genotyped PMBB dataset of 4,078 women with EHR-diagnosed endometriosis using 17 clinical features (S2 Fig). We tested four methods for unsupervised clustering as well as 19 values for the number of clusters (K=2–20) and measured three metrics to empirically choose a clustering method and number of clusters (Figure 1).

**Figure 2.**
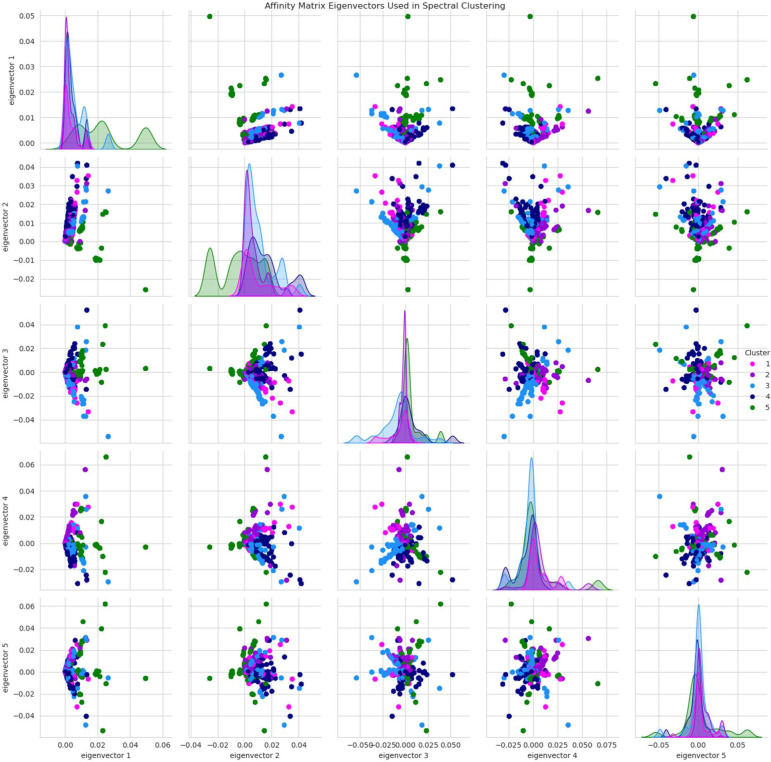
Eigenvector pair plot. Shown are pairwise scatter plots of the first five eigenvectors of the affinity matrix used for spectral clustering, colored by cluster. This five-dimensional eigenvector space was used for clustering. The diagonal shows kernel density estimator plots for each of the five eigenvectors.

Based on these tests, we first eliminated DBSCAN because the inferred number of clusters was 131, a far too complex model to be useful or interpretable. Next, we eliminated hierarchical clustering because the sizes of the resulting clusters were more uneven than the other methods. Spectral clustering and k-means clustering were ultimately more difficult to choose between, but when we focused on the shapes of the distortion curves across the values of K, we observed that k-means lacked an “elbow” to show a clear optimal K value whereas spectral clustering clearly indicated 5 as an ideal K with a local minimum. Thus, we chose spectral clustering with K=5 as our unsupervised subtyping model. The sizes of the final clusters were: (1) 441 – 11%, (2) 686 – 17%, (3) 1,151– 28%, (4) 796 – 20%, and (5) 1,004 – 25%. Figure 2 illustrates the eigenvectors of the affinity matrix which were used for clustering the data points.

### Data-driven cluster characterization

**Figure 3.**
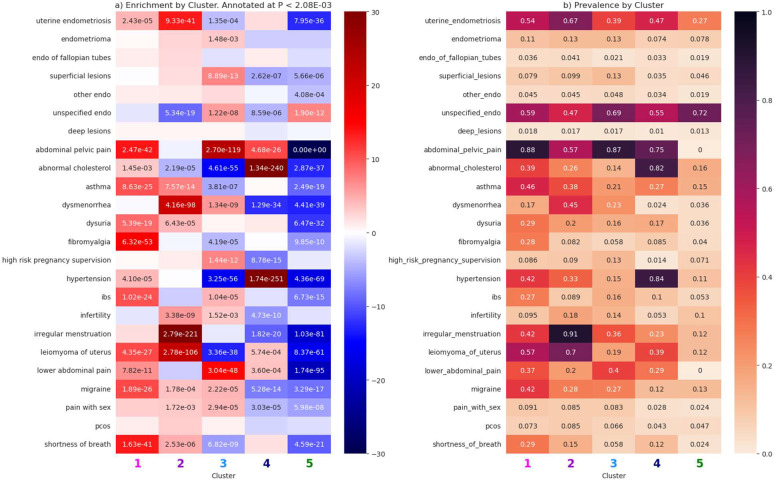
Feature tests for the non-genotyped PMBB training set. Shown are (a) z-scores for the difference in proportion tests, annotated with p-values that are significant and (b) feature prevalence by cluster to provide context for the z-score tests.

After clustering, we aimed to characterize these clusters by observing patterns in clinical presentation (prevalence) amongst the input features. We performed two sets of z-score proportion tests comparing prevalence of each feature between each cluster and the other four clusters in our training set. The first set of tests was performed on the original cluster derivation cohort, and the features included were the 17 input features (symptoms and comorbidities with prevalence > 5%) as well as ICD-defined anatomical subtypes of endometriosis (Figure 3).

Among the five clusters identified in the training set, there were many input features and ICD-based anatomical subtypes with significantly different proportions. To identify distinguishing features between the clusters, we focus on phenotypes which were significantly enriched and had the highest prevalence in that cluster. Cluster one had the highest rates of (and was significantly enriched for) dysuria (Z=8.9), migraine (Z=10.6), IBS (Z=10.3), fibromyalgia (Z=15.3), asthma (10.3), abdominal pelvic pain (Z=13.6), and shortness of breath (Z=13.5). Cluster two had the highest rates of the following significantly enriched traits: dysmenorrhea (Z=21.9), infertility (Z=5.9), irregular menstruation (Z=31.75), leiomyoma of uterus (Z=21.9), and uterine endometriosis defined by ICD-9 617.0* or ICD-10 N80.0* (Z=13.4). Cluster three’s defining features were high risk pregnancy supervision (Z=7.1), superficial lesions defined by ICD-9 617.3* or ICD-10 N80.3* (Z=7.1), and lower abdominal pain (Z=14.6). Individuals in cluster four had highest prevalence of abnormal cholesterol (Z=33.1) and hypertension (Z=33.9), while cluster five was only enriched for unspecified endometriosis defined as ICD-9 617.9* or ICD-10 N80.9* (Z=7.0).

**Figure 4.**
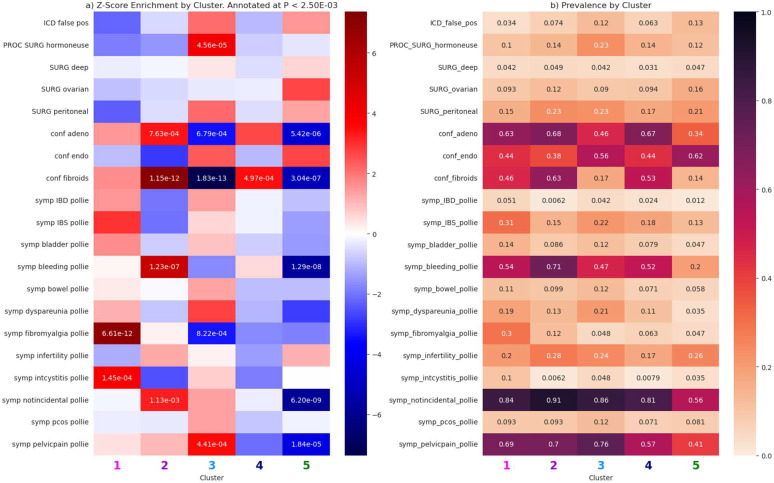
Feature tests for the chart reviewed PMBB dataset. Shown are (a) z-scores for the difference in proportion tests, annotated with p-values that are significant and (b) feature prevalence by cluster to provide context for the z-score tests.

The second set of tests was performed on a subset of endometriosis cases (N=682) from the genotyped PMBB for whom chart reviews were performed by OB-GYN clinical fellows at the University of Pennsylvania Hospital System. The features tested were gold standard confirmed diagnoses (endometriosis, adenomyosis, fibroids, and any ICD false positives), surgical subtypes, hormone use at the time of confirmation procedure, and symptoms identified from a combination of structured data and notes (Figure 4).

Because the size of our chart-reviewed dataset was limited, there were fewer significant tests. For cluster one, the phenotypes which were most significantly prevalent were interstitial cystitis (Z=3.8) and fibromyalgia (Z=6.9). For cluster two, the defining features were confirmed adenomyosis status (Z=3.7), confirmed uterine fibroids (Z=7.1), and severe menstrual bleeding (Z=5.3). Cluster three’s most highly enriched features were pelvic pain (Z=3.5) and hormone use at the time of surgery (Z=4.1). Considering the enriched features for each cluster among the two sets of tests, we defined the following labels for 5 clusters: (1) pain comorbidities, (2) uterine disorders, (3) pregnancy complications, (4) cardiometabolic comorbidities, and (5) EHR-asymptomatic.

### Candidate gene association testing stratified by clusters

We applied the subtype classifications observed in our derivation set to our five genetic association datasets, PMBB, eMERGE, AOU, UKBB, and BioVU. We used a K-nearest neighbors model with k=3 to assign endometriosis cases to the five phenotypes based on the same 17 EHR-derived features (Table 2).

The smallest cluster was the pain comorbidities cluster, with only 14.6% of total endometriosis cases being assigned to this cluster. The EHR-asymptomatic cluster was the largest cluster overall (28.0% of cases). The other three clusters occurred in relatively even proportions in the overall meta-analysis group at 18.5% (uterine disorders), 20.0% (pregnancy complications), and 18.9% (cardiometabolic comorbidities).

**Figure 5:**
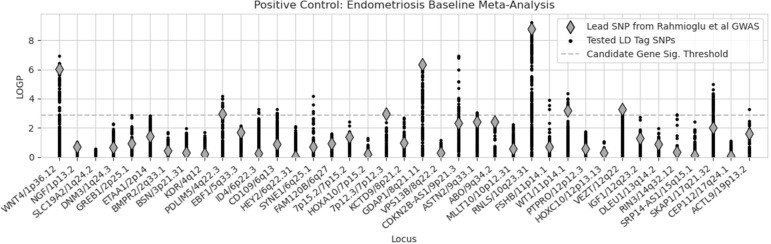
Endometriosis candidate gene positive control. Results for our endometriosis case vs control positive control association tests at each of the 39 known loci. Shown are the lead SNPs from the Rahmioglu et al 2023 GWAS (diamonds) as well their tag SNPs in LD (kb distance < 0.5 Mb and R^2^ > 0.1). X-axis labels are from the known GWAS.

To establish a reference for the expected level of signal replication, we began with a positive control test. We conducted association tests on 39 established genetic locations (autosomes only) known to be linked to endometriosis (Figure 5).

Our positive control test resulted in sixteen replicating loci. Only one was genome-wide significant, *RNLS/10q23.31* (P = 5.79 × 10^−10^, rs4934404:T). Fifteen were significant at a Bonferroni-corrected threshold of 0.05 / 39: *WNT4/1p36.12* (P = 1.20 × 10^−7^, rs2235529:T), *PDLIM5/4q22.3* (P = 6.73 × 10^−5^, rs2452597:G), *ID4/6p22.3* (P = 5.04 × 10^−4^, rs594259:G), *CD109/6q13* (P = 5.41 × 10^−4^, rs4554292:A), *SYNE1/6q25.1* (P = 6.43 × 10^−5^, rs13218956:T), *7p12.3/7p12.3* (P = 1.12 × 10^−3^, rs55909142:T), *GDAP1/8q21.11* (P = 3.68 × 10^−7^, rs10957712:T), *CDKN2B-AS1/9p21.3* (P = 1.13 × 10^−7^, rs10811669:C), *ASTN2/9q33.1* (P = 8.58 × 10^−4^, rs73655468:T), *FSHB/11p14.1* (P = 1.26 × 10^−4^, rs77055031:G*), WT1/11p14.1* (P = 4.44 × 10^−5^, rs2207548:A), *VEZT/12q22* (P = 3.11 × 10^−4^, rs7310833:A), *RIN3/14q32.12* (P = 1.23 × 10^−3^, rs75772533:T), *SKAP1/17q21.32* (P = 9.82 × 10^−6^, rs4794447:C), *ACTL9/19p13.2* (P = 5.29 × 10^−4^, rs35276077:T).

**Figure 6:**
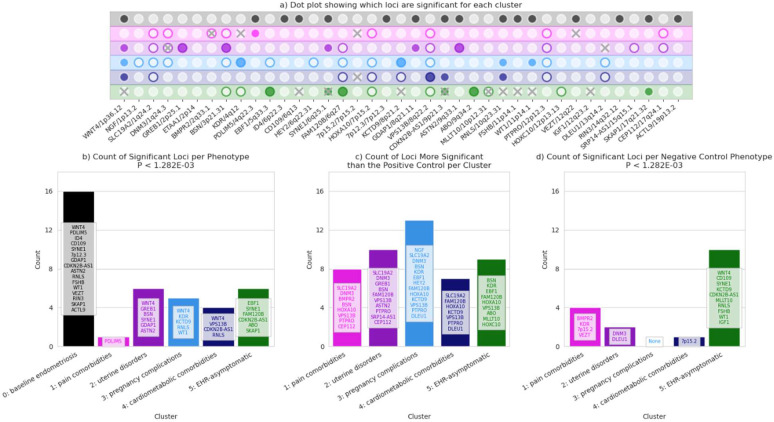
Phenotype-specific association test results. The top panel (a) indicates which known loci were significantly replicated by the positive control and five clusters (filled in dots). The outline of the dots represents whether the cluster had a stronger association than the positive control. An “X” over the dot means that that locus was found to be significant in the negative control for that cluster. The bottom left panel (b) shows the number and names of statistically significant associations for each phenotype. The bottom center panel (c) shows the number of loci for which each phenotype had a more significant association than the baseline. The bottom right panel (d) shows any loci which were significantly associated with the randomly-assigned negative control phenotypes.

To test whether stratifying by clinical presentation allowed for greater resolution in genetic associations, we performed case-control candidate gene association studies for the five phenotypic clusters by meta-analyzing ancestry-stratified summary statistics from five EHR-linked genetic datasets: PMBB, eMERGE, AOU, UKBB, and BioVU. We observe 17 / 39 loci (44%) significantly associating with one or more clusters (Figure 6a,b). Also, for 20 loci, one or more cluster phenotypes yield stronger associations than the positive control despite having smaller sample sizes (Figure 6c). Some loci were significantly replicated in the negative control, indicated by Figure 6d.

The smallest cluster, cluster one, with high rates of pain comorbidities, was significantly associated with one known locus, *PDLIM5*, and it was more significantly associated than the positive control for eight loci, as shown in Fig 6c. The uterine disorders cluster (two) was significantly associated with six loci, *WNT4*, *GREB1*, *BSN*, *SYNE1*, *GDAP1*, and *ASTN2*. Out of the five loci significantly associated with the pregnancy complications cluster (three), two of them were not significantly associated with any other clusters or the positive control: *KDR* and *KCTD9*. Cluster four, enriched for cardiometabolic comorbidities, was significantly associated with four loci, *WNT4*, *VPS13B* (unique to this cluster), *CDKN2B-AS1*, and *RNLS*, the strongest hit from the positive control. *RNLS* was also significantly associated with cluster three. Five loci were significantly associated with the EHR-asymptomatic cluster, and three of those (*EBF1*, *FAM120B*, and *ABO*) had no other associations, even with the positive control. However, two of those five loci (*SYNE1* and *CDKN2B-AS1*) were significantly associated with a negative control test of the same size.

## Discussion

In this study, we aimed to investigate the genetics of heterogeneity in endometriosis by defining data-driven subtypes in women from the non-genotyped PMBB endometriosis population (N=4,078). Unsupervised clustering and statistical enrichment testing across the features, yielded 5 clusters which were labeled as (1) pain comorbidities, (2) uterine disorders, (3) pregnancy complications, (4) cardiometabolic comorbidities, and (5) EHR-asymptomatic. This nuanced phenotyping, which diverges from traditional classifications, allows for a deeper understanding of the pathophysiological variations within endometriosis and highlights the necessity of tailored therapeutic approaches.

After deriving and characterizing the clusters in the non-genotyped PMBB, we used a k-nearest neighbors’ model to transfer the subtypes to the other five EHR-linked genetic datasets, PMBB, eMERGE, AOU, UKBB, and BioVU. Next, we performed ancestry-stratified candidate gene testing for each of the clusters, a positive control, and negative controls using SAIGE. The positive control (overall endometriosis) replicated one genome-wide significant signal (*RNLS*), while 15 more surpassed the Bonferroni threshold. At the Bonferroni significance threshold, the association with RNLS was also significant for two out of five sub-phenotypes: pregnancy complications and cardiometabolic comorbidities. *RNLS* is highly expressed in the heart and contributes to regulating blood pressure ^17^. In genetic association studies, *RNLS* has been previously associated with type 1 diabetes ^18^ and smoking initiation ^19^. Smoking is a known risk factor of endometriosis.

While 62% (41% by the positive control) of previously known GWAS loci were replicated in our study by one or more of the clusters or the positive control, substantial differences in associations across the clusters were observed. For instance, *SYNE1* and *GREB1* showed specific associations with the uterine disorders cluster, suggesting that these genes might play distinct roles in the pathogenesis of these phenotypic presentations of endometriosis. Conversely, the *BSN* gene, although not statistically significant, demonstrated greater significance in the pain, uterine disorders, and pregnancy complications clusters, indicating a possible link to neurovascular or inflammatory mechanisms that could exacerbate these conditions. Cluster five, which was largely asymptomatic in the EHR, was the largest cluster, comprising 56% of UKBB endometriosis patients. It is possible that those assigned to this cluster has unrecorded symptoms in the structured data we accessed. Two well-known endometriosis loci, *SYNE1* and *CDKN2B-AS1* ^13,14^, were significantly associated with both the positive control and the EHR-asymptomatic cluster. However, these loci were also significantly associated with cluster five’s negative control. Additionally, three loci: *EBF1*, *FAM120B*, and *ABO* were uniquely associated with this cluster. These genes have links to several other women’s health conditions: *EBF1* mRNA have been associated with spontaneous preterm birth ^20^, *FAM120B* ^21^ and ABO ^22^ have both been found to be related to ovarian cancer. Notably, endometriosis is known to increase one’s risk of ovarian cancer ^23^.

Subtyping complex diseases, like endometriosis, is crucial for advancing nuanced precision medicine. The findings from our study underscore the utility of EHR as a rich resource for disease subtyping and genetic research. The linkage of detailed clinical data with genetic information enables the identification of phenotype-genotype correlations that are often diluted in broader GWAS analyses. Furthermore, the use of spectral clustering helps elucidate the heterogeneity within endometriosis, providing a framework for understanding the multifaceted nature of the disease and facilitating the development of personalized medicine.

However, it is essential to acknowledge the limitations of our study. One significant constraint was the sample size, which was particularly limited for some of the smaller clusters and for individuals of non-European ancestry. This limitation could potentially introduce bias and affect the generalizability of our findings. Additionally, our study relies on structured electronic health data only, which may not capture the full clinical picture and could be subject to inaccuracies or incomplete records. Last, this genetic association analyses in this study only focused on the candidate genes that are previously known to be associated with endometriosis. This approach might have restricted our ability to discover novel genetic loci potentially relevant to the specific clusters identified. Despite these limitations, our study marks a meaningful advancement in understanding the genetic factors that may contribute to the heterogeneity observed in endometriosis. By focusing on genetic associations gleaned from electronic health records, we offer a novel perspective that could be instrumental in future research and treatment approaches. In conclusion, our research highlights the importance of subtype-specific studies in elucidating the genetic basis of endometriosis. By leveraging the capabilities of EHR-linked biobanks and employing advanced clustering techniques, we pave the way for more targeted and effective approaches to understanding and managing this complex disease.

## Methods

### Datasets used for sub-phenotyping and genetic association

The Penn Medicine Biobank (PMBB) is the University of Pennsylvania’s health system-based biobank which consists of about 250,000 consented participants, with 43,624 of those having imputed genotype data (imputed to TOPMED reference panel) linked with their electronic health record (EHR) history. The PMBB is an electronic health record (EHR)-linked biobank that integrates a wide variety of health-related information, including diagnosis codes, laboratory measurements, imaging data, and lifestyle information, with genomic and biomarker data. The PMBB is one of the most diverse medical biobanks, with approximately 30% of participants being of non-European ancestry. This diversity is crucial for ensuring that research findings are applicable to a broad range of populations. The biobank also benefits from a median of seven years of longitudinal data in the EHR, providing valuable information on participants’ health histories ^24^. For our study, we treated the PMBB as two distinct datasets: those without and those with genotype data. EHR data from the non-genotyped PMBB were used for cluster derivation whereas the genotyped PMBB cohort was used in the genetic analyses.

The Electronic Medical Records and Genomics (eMERGE) network is a National Human Genome Research Institute-funded consortium engaged in the development of methods and best practices for using the electronic medical record as a tool for genomic research. The eMERGE network is a publicly-available dataset with contributions from multiple health systems within the United States which contains about 100,000 participants with linked health records and imputed genomic data (imputed to HRC reference panel) ^25^. The eMERGE consortium validated the hypothesis that clinical data derived from electronic medical records can be used successfully for complex genomic analysis of disease susceptibility across diverse patient populations ^26^. The eMERGE network has shown the efficiency that can result from the use of electronic health record data.

The All of Us (AOU) Research Program is an initiative created by the NIH to recruit demographically diverse individuals to the largest US-based biobank to-date. Recruitment began in 2018, and since then, over 400,000 people have signed up and submitted baseline questions ^27^. 245,388 of them have short-read whole genome sequence data, collectively representing over one billion genetic variants ^28^. Participants’ EHRs are contributed to the AOU data processing center using the Sync for Science platform ^29^, which works with EHR vendors such as Epic and Cerner to collate structured patient data for research use ^30^.

The UK Biobank (UKBB) is a large and comprehensive dataset that provides valuable resources for researchers studying a wide range of health-related topics. The UKBB is a population-based publicly available dataset consisting of about 500,000 UK citizens with EHR data, health survey data, and imputed genotypes. The UK Biobank has performed genome-wide genotyping on all participants using the UK Biobank Axiom Array ^31^. This array directly measures approximately 850,000 variants, and more than 90 million variants are imputed using the Haplotype Reference Consortium and UK10K + 1000 Genomes reference panels.

The Vanderbilt University Biobank (BioVU) is a cohort based in Nashville, TN. It is comprised of EHR records from the Vanderbilt University Medical Center (VUMC) linked with genomic data, developed as described previously ^32^. Briefly, participants were genotyped using a customized Illumina Multi-Ethnic Genotyping chip, and then individuals or variants with high missingness (>2%) were removed. After further filters for revoked consent status or genetic sex discrepancies, whole-genome variants were imputed on the TOPMED Imputation server ^33^ for remaining individuals. The EHR data are stored in a synthetic derivative (deidentified mirror) of over 3 million VUMC patient records which are updated regularly ^34^.

All five of the biobanks mentioned above (PMBB, eMERGE, AOU, UKBB, and BioVU) utilize the Observational Medical Outcomes Partnership Common Data Model (OMOP-CDM) to represent structured EHR data in a harmonized format ^35^. For this study, we utilized women with ICD-diagnosed endometriosis in the non-genotyped PMBB cohort (N_endo_ = 4,078) as the derivation dataset for the clinical subtypes. For deeper characterization of our subtypes, we performed chart-reviews on 682 randomly selected endometriosis cases from the genotyped PMBB. Then, we meta-analyzed women from the genotyped PMBB (N = 20,697, N_endo_ = 1,198), six non-pediatric sites within the eMERGE network (N = 51,800, N_endo_ = 2,243), the AOU research program (N = 108,098, N_endo_ = 2,126), UKBB (N = 261,824, N_endo_ = 4,451), and BioVU (N = 34,072, N_endo_ = 1,097) to form our main genetic analysis test set (N = 466,261, N_endo_ = 12,350).

Genetic ancestry labels for UKBB were determined based on self-reported ancestral background. Each of the other biobanks projected their samples onto the thousand genomes reference population and performed clustering to assign genetically inferred ancestry labels corresponding to those from the thousand genomes project ^36^. We restricted our genetic association analyses to the groups which had substantial sample sizes, which were those with high similarity the AFR and EUR thousand genomes superpopulations. We will refer to those groups using AFR and EUR from here on out.

### Extraction of endometriosis-related clinical features

Patients with endometriosis have heterogeneous clinical presentations; there are a wide variety of associated symptoms, risk factors, and comorbidities. We first determined participants’ case-control status of endometriosis using structured EHR data: ICD-9 and ICD-10 billing codes 617 and N80, respectively. Then for endometriosis cases, we determined whether each individual had a history of endometriosis-related clinical features. In total, we extracted 39 ICD-based features (Table S1): 9 ICD-based anatomical subtypes, 14 comorbidities, 8 symptoms, and 8 pregnancy-related phenotypes. We selected only symptoms, comorbidities, and pregnancy-related conditions for clustering, removing the 9 anatomical subtypes to be used downstream in cluster characterization. We further restricted these conditions to those with a prevalence amongst endometriosis cases in the subtype dataset of at least 5%, leaving us with 17 features for the clustering analysis (S1 Fig).

### Unsupervised clustering

We tested four popular methods for unsupervised clustering: spectral clustering, density-based spatial clustering of applications with noise (DBSCAN), hierarchical agglomerative clustering, and k-means clustering. Spectral clustering identifies clusters by decomposing a dataset’s affinity matrix into its eigenvectors and then clustering in the eigenvector space using QR clustering algorithm ^37,38^. DBSCAN is an algorithm which identifies dense regions of data points to discover clusters ^39^. Hierarchical agglomerative clustering is an unsupervised classification method that uses a pairwise distance matrix to iteratively merge nearby points together ^40^. K-means clustering randomly initializes centroids for each cluster and then alternates between assigning data points to their nearest centroid and adjusting the centroids until convergence ^41^.

In addition to choosing an algorithm, a common struggle with unsupervised clustering is choosing a target number of clusters in a non-arbitrary way. We used several empirical metrics for this: silhouette score, distortion score, and a metric we developed to represent the “evenness” of clusters. The silhouette score is a metric which considers both intra- and inter-cluster distances to assess tightness within a cluster and distance between clusters; higher silhouette scores indicate better quality clusters. The distortion score is the sum of squared errors with respect to the centroid of each cluster, thus it is desired to minimize distortion. Our evenness metric, optimized by minimization, was defined as the fractional difference between the size of the largest and smallest clusters. We measured these metrics across tests for 2–20 clusters for each of the four clustering methods (except for DBSCAN which automatically infers the optimal number of clusters).

### Characterization of unsupervised clusters

After identifying clinical clusters within our observation dataset, our objective was to delineate their characteristics. We performed two-population z-score proportion tests ^42^ to determine if the rates of input conditions were significantly different on a cluster-vs-other-clusters basis. For our training set (the non-genotyped PMBB, N_endo_ = 4,078), we examined two sets of features for the z-score tests: the 17 input features as well as ICD-based anatomical subtypes of endometriosis including adenomyosis, endometrioma, superficial lesions, and deep lesions (S2 Table). We performed a second set of z-score proportion tests on a chart-reviewed dataset of 682 genotyped PMBB patients with endometriosis ICD codes. The chart-review variables collected by OB-GYN clinical fellows at the University of Pennsylvania included gold standard diagnoses of endometriosis and adenomyosis as well as chart-abstracted symptoms, comorbidities, and surgical phenotypes (S3 Table). By considering the cluster-specific differences in these EHR-derived features among the two datasets (training set and chart review set), we observed patterns in clinical presentation. Based on these patterns, we assigned labels to each cluster.

### Cluster-stratified candidate gene association testing

To identify genetic heterogeneity among the varied clinical presentations of endometriosis, we performed cluster-stratified, ancestry-stratified candidate gene association studies. Using PLINK 2.0 ^43^, we extracted single nucleotide polymorphisms (SNPs) in LD (kb distance < 0.5 Mb and R^2^ > 0.1) with 39 autosomal lead SNPs reported in the most recent endometriosis GWAS^14^. LD was computed based on the thousand genomes reference panel ^36^. Cluster phenotypes were assigned for PMBB, eMERGE, and AOU using a K-Nearest neighbors’ classifier ^44^ with K=3 on the same 17 ICD-based features. For each study, we employed a linear mixed model regression method employed in SAIGE ^45^ to test for associations between genotypes and case-control status. Cases were females with endometriosis from one cluster and controls were biological females with no ICD history of endometriosis. In the regression models we included the first four principal components, age, and batch indicators (eMERGE only) as covariates. The ancestry-stratified results of these studies were then meta-analyzed using Plink 1.9 ^46^ for each of the cluster-phenotypes. Because multiple genetic ancestry groups were included, we chose a random-effects meta-analysis, which is more robust to heterogeneity ^47^.

Using the same association testing methodology, we tested a positive control and negative control. The positive control baseline was overall endometriosis (cases from all clusters combined) to identify how many known loci we were able to replicate. The negative control was performed for each of the clusters to see whether we were powered to detect associations regardless of signal coming from the identified subtypes. Endometriosis cases were randomly assigned to one of the five clusters so that the five meta-analyses for the negative control had the same numbers of cases and controls as in the regular analyses.

## Figures and Tables

**Table 1.** Description of Datasets. Cohort sample size and average age of cases and controls for the datasets used in this analysis. Age was considered the age as of when the EHR data were collected.

Dataset	Endometriosis	N (AFR / EUR)	Mean Age (SD)
Cluster Derivation Set:			
Non-Genotyped PMBB	Cases	4,078 (NA)	49.9 (13.3)
Genetic Association Sets:			
AOU	Cases	2,126 (542 / 1,584)	52.2 (12.8)
	Controls	108,099 (31,435 / 76,664)	56.8 (16.8)
eMERGE	Cases	2,243 (353 / 1,890)	59.9 (14.6)
	Controls	49,557 (9,934 / 39,623)	59.7 (23.4)
PMBB	Cases	1,198 (562 / 636)	54.2 (12.9)
	Controls	19,493 (6,524 / 12,969)	60.0 (17.8)
UKBB	Cases	4,541 (112 / 4,429)	51.5 (7.5)
	Controls	257,283 (4,524 / 252,759)	56.6 (8.0)
BioVU	Cases	1,097 (260 / 837)	47.2 (12.5)
	Controls	32,975 (6,376 / 26,599)	53.3 (18.1)
Meta-Analysis Totals:			
META	Cases	12,350 (2,079 / 10,271)	53.1 (11.5)
	Controls	466,261 (58,543 / 407,718)	56.8 (14.0)

**Table 2.** Clusters by dataset. Counts and proportions of endometriosis cases in each cluster by dataset, along with the faction of cases for each subtype.

Dataset	Pain Comorbidities	Uterine Disorders	Pregnancy Complications	Cardiometabolic Comorbidities	EHR-Asymptomatic
Cluster Derivation Set:					
Training	441 (10.8%)	686 (16.8%)	1,151 (28.2%)	796 (19.5%)	1,004 (24.6%)
Genetic Association Sets:					
AOU	713 (21.8%)	690 (21.1%)	723 (22.1%)	783 (23.9%)	362 (11.1%)
eMERGE	495 (22.1%)	505 (22.5%)	382 (17.0%)	709 (31.6%)	152 (6.8%)
PMBB	200 (16.7%)	222 (18.5%)	273 (22.8%)	366 (30.6%)	137 (11.4%)
UKBB	231 (5.1%)	607 (13.4%)	842 (18.5%)	285 (6.3%)	2,576 (56.7%)
BioVU	165 (15.0%)	262 (23.9%)	255 (23.2%)	189 (17.2%)	226 (20.6%)
Meta-Analysis Totals:					
META	1,804 (14.6%)	2,286 (18.5%)	2,475 (20.0%)	2,332 (18.9%)	3,453 (28.0%)

## Abbreviations

AOU: All of Us Biobank
BioVU: Biobank of Vanderbilt University
eMERGE: electronic medical record and genomics network
EHR: electronic health record
GWAS: genome-wide association study
ICD: international classification of diseases
PMBB: Penn Medicine biobank
UKBB: United Kingdom biobank
